# An Efficient Adaptive Angle-Doppler Compensation Approach for Non-Sidelooking Airborne Radar STAP

**DOI:** 10.3390/s150613121

**Published:** 2015-06-04

**Authors:** Mingwei Shen, Jia Yu, Di Wu, Daiyin Zhu

**Affiliations:** 1 College of Computer & Information Engineering, Hohai University, Nanjing 211100, China; E-Mail: yujiahhu@126.com; 2 Key Laboratory of Radar Imagine and Microwave Photonics (Nanjing University of Aeronautics and Astronautics), Ministry of Education, Nanjing 210016, China; E-Mails: wudi82@nuaa.edu.cn (D.W.); zhudy@nuaa.edu.cn (D.Z.)

**Keywords:** space-time adaptive processing, sparse reconstruction, adaptive angle-Doppler compensation, clutter suppression

## Abstract

In this study, the effects of non-sidelooking airborne radar clutter dispersion on space-time adaptive processing (STAP) is considered, and an efficient adaptive angle-Doppler compensation (EAADC) approach is proposed to improve the clutter suppression performance. In order to reduce the computational complexity, the reduced-dimension sparse reconstruction (RDSR) technique is introduced into the angle-Doppler spectrum estimation to extract the required parameters for compensating the clutter spectral center misalignment. Simulation results to demonstrate the effectiveness of the proposed algorithm are presented.

## 1. Introduction

Space-time adaptive processing (STAP) [1,2] performs two-dimensional space and time adaptive filtering to suppress colored interferences such as clutter and jammer in airborne radars. In recent years, the STAP technology research has gradually turned from sidelooking array radars (SLAR) to various other radar array configurations. As a consequence, clutter dispersion mitigation has become a crucial issue for the detection of slowly-moving ground targets. The non-stationary nature of non-SLAR clutter limits the practical implementation of the standard STAP approach, which relies for covariance estimation on secondary data obtained from adjacent range cells. If we directly estimate the clutter covariance matrix using adjacent range cells, a severe degradation in detection performance may occur due to the range dependence of the clutter spectrum, particularly for short-range clutter.

A number of methods to increase secondary data homogeneity have been developed [1,3,4]. The Doppler warping (DW) and high order DW methods try to align the Doppler centroid of the main-lobe clutter of the second data towards the test cell in the angle-Doppler plane. The angle Doppler compensation (ADC) method [4] tries to align the clutter spectral centers (SCs) at different ranges to that of the test cell. Assuming a precise knowledge of the configuration parameters, the registration-based compensation (RBC) method [5] is proposed to register the clutter ridges of the training samples with the test cell by performing appropriate transformations. However, the resulting performance of these compensation algorithms is dominated by the accuracy of configuration parameters, which are always unknown in practice. Based on the assumption that the weight vector varies linearly, the derivative-based updating (DBU) technique [6] achieves favorable performance for some non-SLAR configurations. However, the DBU imposes a considerable computational burden due to its doubling of the covariance matrix dimensions. In reference [7], the adaptive angle-Doppler Compensation (AADC) algorithm, which is able to extract the compensating parameters from the data themselves, is fully adaptive and rather robust for both non-SLAR and bistatic STAP applications [8,9,10]. However, this technique is computationally costly in estimating the whole clutter angle-Doppler spectral trajectory with range.

In order to save computational load, an improved AADC implementation strategy is proposed in this paper. Firstly, a novel clutter spectrum angle-Doppler location estimation method based on reduced-dimension sparse reconstruction [11] is designed to extract the exact knowledge of the clutter angle-Doppler trajectory centers. Then, the clutter spectral center (SC) can be efficiently compensated over range. It is shown that the SC can be estimated with good accuracy from the data themselves. This makes it possible to obtain an effective technique for non-SLAR STAP, which is fully adaptive and rather robust. Moreover, the computational complexity can be dramatically reduced.

The rest of this paper is organized as follows: in Section 2, we first analyze the range dependence clutter dispersion of non-SLAR, and then introduce the principle of conventional AADC. The proposed new approach for parameter estimation in AADC is introduced in detail in Section 3. The overall performance of the conceived strategy is assessed with simulated data in Section 4. Finally, a brief conclusion is given in Section 5.

## 2. Properties of Non-SLAR Clutter

### 2.1. Signal Model

The STAP system under consideration is a pulsed-Doppler radar residing on an airborne platform. The radar antenna is a uniform linear array which consists of *N* elements with the element spacing *d* being half of the wavelength λ. The aircraft moves with constant velocity *v* at an altitude *H*.

**Figure 1:**
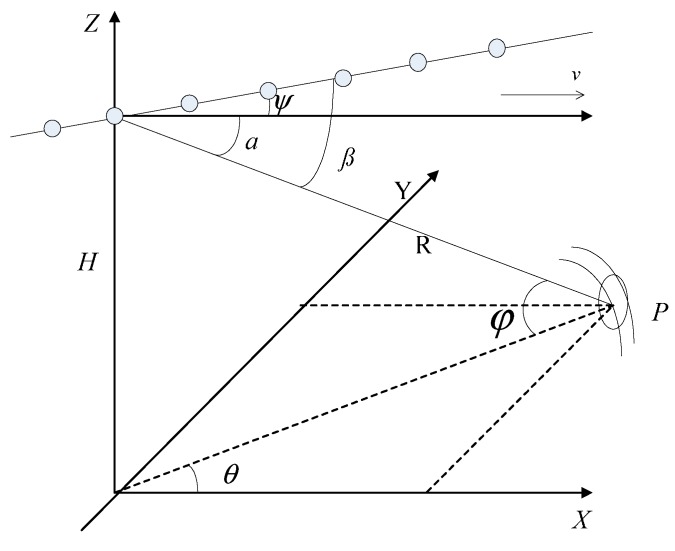
Non-SLAR geometry.

Without loss of generality, the non-SLAR geometry is shown in Figure 1. The coordinate system is assumed that, the x-axis is aligned with the flight direction, the point *P* stands for one scatterer in the scenario of interest, and ψ is the yaw angle between the array and the flight direction. The azimuth angle and elevation angle are represented by θ and φ, respectively. Suppose the radar transmits *K* pulses during the coherent processing interval (CPI). According to the radar platform geometry introduced in Figure 1, the received clutter data for the *l*th range gate can be organized into a space-time snapshot $X_{l}\in {\text{C}}^{NK\times 1}$, and can be expressed as:

(1) $$X_{l}=\sum_{i=1}^{N_{c}}{\text{σ}}_{i}S_{i}+N_{l}$$

where *N_c_* is the number of independent clutter patches, ***S_i_*** indicates the normalized space-time steering vector corresponding to the *i*th patch, **σ*_i_*** describes the complex gain which is proportional to the square-root of the clutter patch radar cross section (RCS), and ***N_l_*** represents the received noise.

In the case of non-SLAR, the clutter trajectory in angle-Doppler domain [1] can be given by:

(2) $$f_{\text{c}}^{2}-2f_{c}\cos\beta \cos\psi +\cos^{2}\beta =\sin^{2}\psi \cos^{2}\theta$$

Where $f_{c}=\frac{f_{d}\lambda }{2v}=\cos\alpha$, $f_{d}=\frac{2v}{\lambda }\cos\alpha$.

**Figure 2:**
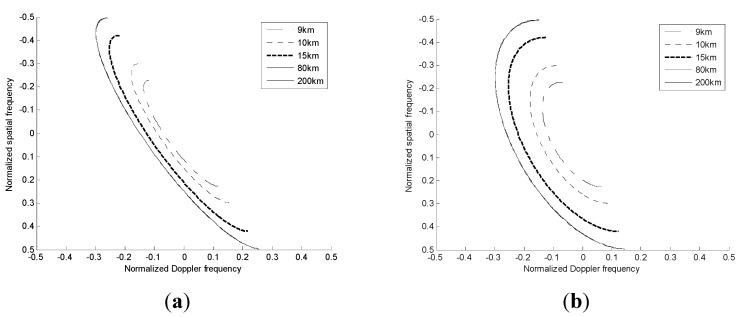
Angle-Doppler trajectories of different range cells. (**a**) ψ = 30°; (**b**) ψ = 60°.

For SLAR, we know that the Doppler frequency is a linear function of the azimuth spatial frequency. Thus, the clutter returns of SLAR can be considered to be range independent. However, in the non-SLAR case, *i.e*., 0° < ψ ≤ 90°, the Doppler frequency is not only a linear function of the azimuth spatial frequency, but also a non-linear function of the elevation spatial frequency. Thus, the non-SLAR clutter exhibits range dependence, which is illustrated in Figure 2.

The clutter trajectories corresponding to different ranges are depicted in Figure 2. It is apparent that the motion of the platform induces a non-stationary behavior of the clutter spectral traces with range. Therefore, the clutter data are not independent and identically distributed (IID) at all ranges. If the training data are applied to estimate the clutter covariance matrix, the range dependence of the clutter ridge will consequently lead to a degraded STAP clutter suppression performance.

### 2.2. Overview of the AADC Approach

The AADC method involves adaptively compensating each space-time snapshot to the estimated peak clutter response of a reference cell. The implementation of space-time data modulation to align two dimensional clutter characteristics over the training range interval was described in [8,10]. Then, the peak angle-Doppler response of a given snapshot can be compensated to a suitably chosen reference range cell before obtaining the interference covariance matrix.

In reference [10], the AADC method uses a sub-aperture smoothing procedure to acquire the required number of space-time data samples for estimating the sample covariance matrices. Although this can overcome the small sample size problem by exploiting the space-time structure of the steering vector together with the uncorrelated nature of the components of the interference covariance matrix, the computational load can be a very heavy burden to estimate the clutter spectrum for each range gate using the minimum variance (MV) estimator [12].

## 3. The Implementation of EAADC

**Figure 3:**
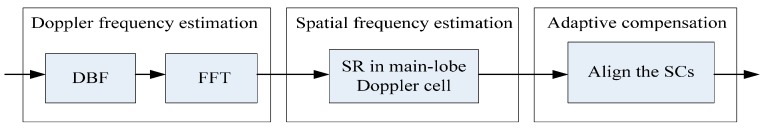
The EAADC flowchart.

The above statement shows that the non-SLAR range dependence clutter dispersion leads to the two-dimensional clutter spectrum broadening seriously, which decreases the subsequent STAP performance for slow-moving target detection. In this paper, we propose a novel approach to significantly reduce the complexity of the compensation parameter estimation used in AADC. The implementation of the proposed EAADC strategy is summarized in the following steps and sketched in Figure 3.

### 3.1. Estimation of Main-Lobe Clutter Doppler Frequency

Since it is well known that the Doppler frequency of main-lobe clutter is determined by the round-trip modulation of the transmitter (TX) and receiver (RX) antenna beam direction, and the pulse number *K* is much greater than the array elements *N*, thus the received sum beam can be derived via digital beam forming (DBF) to estimate the Doppler frequency of the main-lobe clutter Doppler frequency. For notional convenience, the received data *X_l_* for range gate *l* can be reshaped into a *N* × *K* matrix as:

(3) $$X_{l}^{0}={\left[S_{l\_1}\;S_{l\_2}\cdots \;S_{l\_K}\right]}_{N\times K}$$

where ***S****_l_i_* represents the spatial snapshot vector from the *N*-element array for the *i*th pulse of a *K*-pulse CPI. Given the receiving sum beam weight vector ***W***_Σ_, the output of the receiving sum beam can be described as:

(4) $$\Sigma_{l}=W_{\text{Σ}}X_{l}^{0}$$

Therefore, the sum beam output can be transformed to the Doppler domain via a one-dimension Fourier Transform, which can be implemented as:

(5) $$D_{l}=\Sigma_{l}F_{D}^{H}=[D_{l\_1}\;D_{l\_2}\cdots \;D_{l\_K}]$$

where the superscript *^H^* denotes the conjugate transpose operation, ***F****_D_* comprises the Fourier Transform weights, and ***D****_l_i_* represents the output of the *i*th Doppler cell. As a consequence, the main-lobe clutter Doppler frequency can be determined by the maximum value of ***D****_l_*.

### 3.2. Estimation of the Main-Lobe Clutter Spatial Frequency

If we still utilize FFT to obtain the angle spectrum in the spatial domain, the angle-Doppler spectrum will suffer from a series of problems such as main-lobe broadening, and high side-lobe leakage, which consequently leads to a poor estimation of the spatial frequency. Therefore, the RDSR technique [11] can be used to obtain the high resolution spatial spectrum.

Toward the end of estimating the spatial frequency of main-lobe clutter, only the array output corresponding to the main-lobe clutter Doppler cell should be transformed to the spatial domain. Therefore, the angle spectrum can be achieved:

(6) $$\begin{array}{l} {\hat{\sigma }}_{\text{d}\max\_l}=\arg\min{\left\|{\text{σ}}_{\text{d}\max\_l}\right\|}_{1}\\ \text{s.t. }\left\|A_{l\_\max}-{\text{ψ}}_{i}{\text{σ}}_{\text{d}\max\_l}\right\|\leq {\text{ε}}_{i} \end{array}$$

where ***A****_l__*_max_ is the element-Doppler data for the maximum Doppler cell of the *l*th range gate, **σ**_dmax_*l*_ represents the estimated clutter distribution response in spatial domain, ψ*_i_* is an overcomplete basis representation in terms of all possible sources locations, and ε*_i_* is the error allowance. Compared with two-dimensional sparse reconstruction (SR) in the angle-Doppler domain [13,14,15], the computational load of our method is significantly reduced since the computation-cost SR is only applied in the angle domain.

It is noted that the proposed approach only requires the knowledge of the spectral center at each range cell. Thereby, the single parameter can be estimated by applying the RDSR to the main-lobe Doppler cell. This greatly simplifies the estimation problem and makes the proposed technique potentially more suitable for practical application.

**Figure 4:**
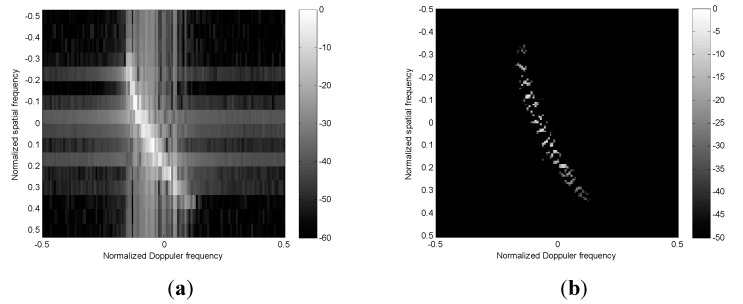
The clutter angle-Doppler spectrum image with a 10.5 km slant distance. (**a**) 2D FFT; (**b**) Spatial RDSR.

To evaluate the performance of RDSR in the spatial domain, the angle-Doppler spectrum obtained by using RDSR in each Doppler cell is shown in Figure 4b, where we assume *N* = 16, *K* = 128, ψ = 30°, and the clutter distance is 10.5 km. For comparison purposes, the spectrum obtained via 2D FFT is also provided in Figure 4a. It is clear that the spatial RDSR image has much better resolution and lower side-lobe level than that of 2D FFT, which will improve the accuracy of estimating the SC location of main-lobe clutter. However, due to the spectrum discontinuity of RDSR in the spatial domain, the spectral center frequency extracting method proposed in reference [11] is also employed to determine the maximum value of the angle spectrum.

### 3.3. Adaptive Compensation of the Spectral Centers

The weight of the STAP approach is calculated based on the covariance matrix, which is estimated using adjacent range cells as training samples. However, the non-stationary nature of non-SLAR clutter as shown in Figure 2 decreases the performance of STAP. A number of contributions have been reported where the clutter range dependence problem is largely addressed, and the AADC is an effective approach to directly align peak angle-Doppler responses or SCs over range. Therefore, the range dependence SCs of the 2D spectrum now can be effectively removed, since the Doppler and spatial frequency of main-lobe clutter have already been estimated in Sections 3.1 and Sections 3.2, respectively.

The adaptive compensation scheme applied to the *l*th range cell secondary data can be described as follows:

(1) We assume that the estimated SC of the reference range cell is (${\text{f}}_{s,0}$, ${\text{ρ}}_{s}$), and the SC of the *l*th range cell is (${\text{f}}_{s,l}$, ${\text{f}}_{d,l}$). Hence, the displacements in terms of spatial frequency and Doppler frequency can be determined as:

(7) $$\Delta f_{d,l}={\text{f}}_{d,l}-{\text{f}}_{d,0}$$

(8) $$\Delta f_{s,l}={\text{f}}_{s,l}-{\text{f}}_{s,0}$$

(2) Both the Doppler and angle shifting to compensate for the SC migration can be achieved by applying a complex linear phase taper over both the Doppler and angle dimensions, respectively, which can be given by:

(9) $$C_{l}=T_{s,l}X_{l}^{0}T_{t,l}$$

where **T***_t,l_* represents the vector taper in the Doppler domain, and **T***_s,l_* is the vector taper in the spatial domain. They can be expressed as:

(10) $$T_{t,l}={\left[\begin{array}{cccc} 1 & \exp(j2\pi \Delta f_{d,l}/f_{r}) & \cdots & \exp(j2\pi (K-1)\Delta f_{d,l}/f_{r}) \end{array}\right]}^{T}$$

(11) $$T_{s,l}=[\begin{array}{cccc} 1 & \exp(j2\pi \Delta f_{s,l}) & \cdots & \exp(j2\pi (N-1)\Delta f_{s,l}) \end{array}]$$

where the superscript *^T^* denotes the transpose operation. As expected, by applying the aforementioned implementations to the secondary data before covariance estimation, the STAP performance can be significantly improved owing to the reduction of clutter dispersion. To evaluate the effectiveness of the proposed strategy, when considering the performance of mitigating the clutter dispersion we assume that the adaptive angle-Doppler compensation techniques (AADC and EAADC) are applied in conjunction with a reduced-dimension (RD) STAP approach. The detailed performance analysis will be given in Section 4.

## 4. Performance Analysis

This section is devoted to the performance assessment of the proposed scheme using simulated data. In the simulation, we consider a non-sidelooking airborne early warning radar with *N* = 16 antenna elements and *K* = 128 pulses in one CPI. The crab angle between the array and the flight direction is ψ = 30°. The simulation parameters are listed in Table 1.

**Table 1:** Simulation Parameters for AEW radar.

PRF	5000 Hz
Bandwidth	5 MHz
Array Element Number	16
Platform Velocity	130 m/s
CPI Pulse Number	128
Crab angle	30°
Platform Height	8000 m
Element spacing and wavelength ratio	1/2

Specifically, the application of the EAADC technique in conjunction with a RD STAP is investigated and the obtained improvement factor (IF) results, given as the ratio of output SINR to the input SINR, are presented and analyzed. Among the many RD STAP algorithms proposed in the literature [1,16], the well-established Extended Factored STAP (EF-STAP) technique is considered [16].

**Figure 5:**
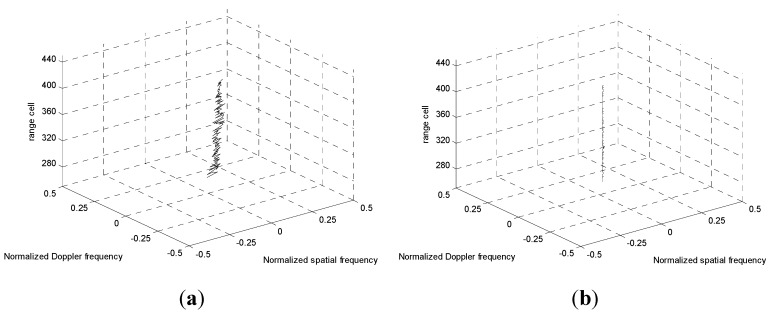
SCs migration over range. (**a**) Before compensation; (**b**) After Compensation.

The SCs migrations over range are depicted in Figure 5. From Figure 5a we can observe that the clutter SCs before compensation are misaligned over range due to the non-SLAR configuration. It is given that the range cells from 280 to 430 are compensated based on the reference SC of the 350th range cell. Therefore, the clutter spectrum SCs after realignment using the new EAADC are depicted in Figure 5b. It is apparent that the SCs of the spectral traces are exactly co-located.

**Figure 6:**
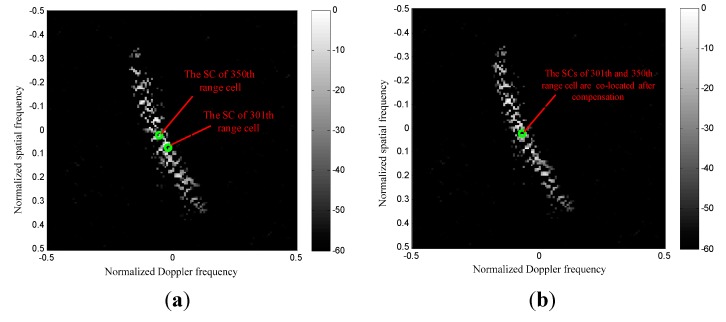
Spectrum of different range cells. (**a**) Without compensation; (**b**) Using EAADC approach.

The results in Figure 6a,b are shown for the spectrum of two different range cells before and after SC compensation, respectively. By applying the EAADC compensation strategy to the secondary data, the clutter spectrum dispersion reported in Figure 6b is greatly reduced. As a consequence, no dispersion should occur around the Doppler-angle bin corresponding to the reference range cell. Then the IF notch obtained with the EAADC will be narrower, which yields a significant clutter suppression advantage.

**Figure 7:**
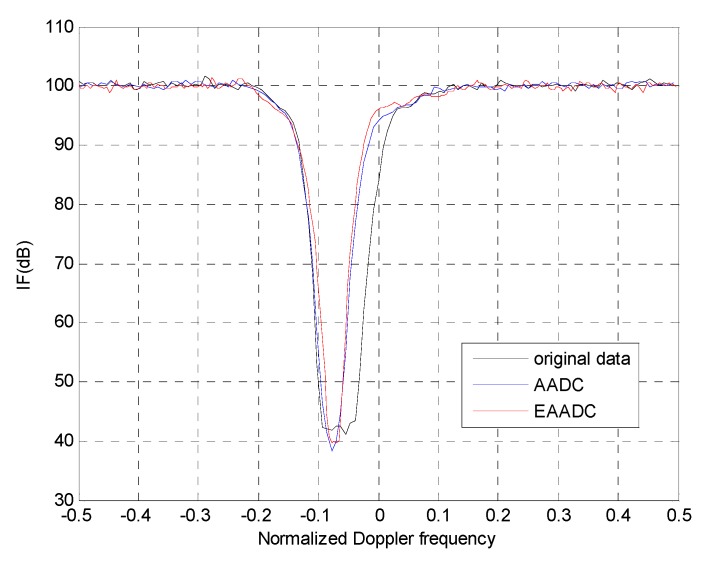
IF of 3DT after the application of the compensation techniques.

IF is another common metric to evaluate the capability to compensate the clutter SCs misalignment. Figure 7 presents the IF curves obtained after applying the AADC and EAADC approach to the secondary data, where the 350th range cell is selected as the reference cell. Apparently, it yields a narrower IF notch since the range dependence clutter dispersion has been significantly mitigated after main-lobe compensation. Consequently, the IF of the pre-processing using the AADC approach is increased about 13.59 dB with respect to the EF-STAP without data pre-processing. Specifically, it is to be observed that the IF curve obtained with the EAADC yields about 1.42 dB higher in the notch area than that obtained with AADC. The additional improvement is only because the EAADC can estimate the parameters more accurately than the MV estimator which suffers lost degrees of freedom due to the sub-aperture smoothing.

**Figure 8:**
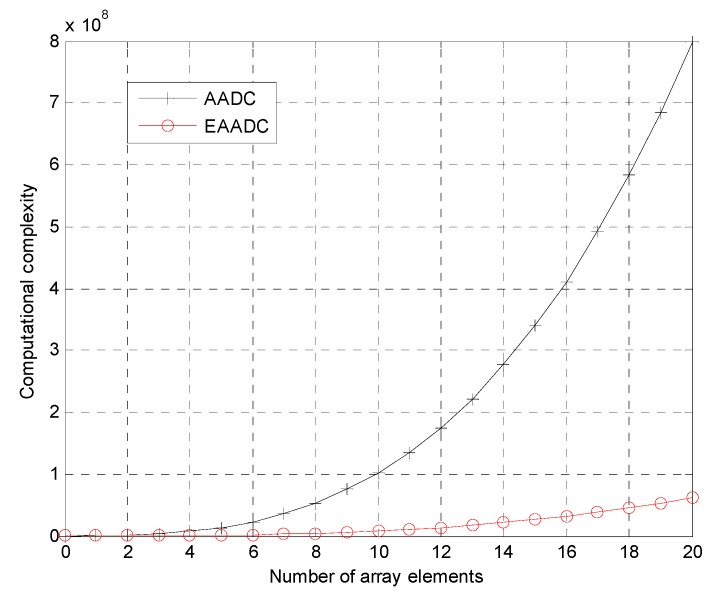
The main computation complexities of AADC and EAADC.

Finally, the computational complexity is analyzed. The computational workload of our method is less than the conventional AADC. A more comprehensive study of the operation counts is expressed in terms of multiplier operations. For the MV estimator used in the AADC, the dimension of the smoothed covariance is *N*′*K*′ × *N*′*K*′ where *N*′ is the number of the sub-array elements and *K*′ is the number of the sub-CPI pulses. For a typical scenario, given *N*′ = 3/5*N* and *K*′ = 3/5*K*, thus the computational complexity of AADC at each range cell is about *O*[9/25*NK*)^3^ + (27/125*NK*^2^)(2/5*N* + 1)(2/5*K* + 1)]. In contrast, the EAADC obtains the compensation parameters using RDSR, which is implemented by applying the SR corresponding to the array output of the maximum Doppler bin. The computational complexity of RDSR is about $O\left[{\text{ρ}}_{\text{s}}^{3}N^{3}\right]$ per iteration where ρ*s* is the angle resolution scale, and it requires about 15 iterations to carry out the problem [17]. Therefore, the total computational complexity of EAADC is about $\left(N+K\log_{2}K)+O[15{\text{ρ}}_{\text{s}}^{3}N^{3}\right]$. In our simulation, the angle resolution scale is set to be ρ*_s_* = 8. As a consequence, it can be found that the main computational complexity of EAADC is much smaller than that of the conventional AADC. Moreover, given *K* = 128, the main computational complexities of the AADC and EAADC are calculated according to the number of array elements *N*. The corresponding results are shown in Figure 8. It can be observed that the increasing trend of the main computational complexity of the proposed method is also much smaller than that of AADC. Therefore, the proposed EAADC method has many more advantages in computational complexity.

## 5. Conclusions

This work proposed an improved EAADC algorithm to mitigate the range dependence clutter dispersion for non-SLAR STAP. The requisite spatial frequency for realigning the spectral center is calculated using the RDSR method instead of the typical MV estimator, so that the computational complexity can be reduced dramatically. The effectiveness of the proposed method has been demonstrated against simulated data which showing that this strategy is able to further reduce the non-SLAR geometry-induced loss on STAP with respect to the previous AADC technique. Additionally, the proposed method is more suitable for real-time processing due to its high computational efficiency.
